# Electric-Field-Based Guidance for Percutaneous Catheter Vessel Crossing

**DOI:** 10.3390/s22134928

**Published:** 2022-06-29

**Authors:** Mamadou Diallo, Clemens Eder, Daniel Brasier, Sorin Popa, Robert Dickinson

**Affiliations:** 1 Pathfinder Medical, London SW11 3TZ, UK; mamadou@pathfindermed.com (M.D.); clemens@pathfindermed.com (C.E.); dan@pathfindermed.com (D.B.); sorin@pathfindermed.com (S.P.); 2 Department of Bioengineering, Imperial College, London SW7 2AZ, UK

**Keywords:** anastomosis, fistula, catheter, intravascular, electric field sensor

## Abstract

Percutaneous procedures to divert blood flow from one blood vessel to another can be performed with intravascular catheters but demand a method to align a crossing needle from one vessel to another. Fluoroscopic imaging alone is not adequate, and it is preferable to have a sensor on one catheter that detects the correct alignment of an incoming needle. This can be implemented by generating dipole electric fields from the crossing catheter which are detected by a receiving catheter in the target vessel and, thus, can calculate and display the degree of alignment, permitting the operator to rotate the crossing catheter to guarantee alignment when deploying a crossing needle. Catheters were built using this concept and evaluated in vitro. The results show that accurate alignment is achieved, and a successful crossing can be made. The concept is being further developed for further clinical evaluation.

## 1. Introduction

The treatment of some diseases requires the diversion of blood flow from one vessel to another. Procedures that require this include bypass re-entry in chronic total occlusion [1] and arterio-venous fistula (AVF) creation for dialysis [2]. Although this can be achieved surgically, there are considerable clinical advantages in performing the procedure percutaneously using intravascular catheters [3]; currently, the failure rate for surgical AVFs after one year is as high as 40% [2,4]. In the percutaneous procedure, a catheter which houses a preformed, hollow needle is inserted into a vessel, and the needle is then deployed out of the catheter to penetrate the vessel wall and into the neighbouring target vessel. Once the needle has crossed between the vessels, a guide wire can be inserted through the needle into the target vessel, and this guide wire is used in the subsequent deployment of devices, such as stent grafts, to create a channel between the vessels. A major challenge of this procedure is ensuring the catheter is rotated to the appropriate position so that when the needle is deployed, it enters the target vessel. The standard fluoroscopic visualisation, a 2D projection image, is not usually adequate for this, and so devices have been developed to aid the alignment. These use a variety of alignment techniques such as radio-opaque markers [5,6] or intravascular ultrasound imaging mounted on the delivery device [7,8]. Optical imaging techniques, such as OCT, do not have the range to image neighbouring vessels through opaque intervening tissue. This paper reports on a novel device to achieve the alignment; the crossing catheter generates an electric field that is detected by a target device inserted into the target vessel [9]. Details of the device design and the bench validation of the device in phantoms are presented, showing how the targeting accuracy of the device meets the clinical requirements and how a real-time display of the alignment is presented to the clinician.

## 2. Materials and Methods

### 2.1. System Design

**Figure 1:**
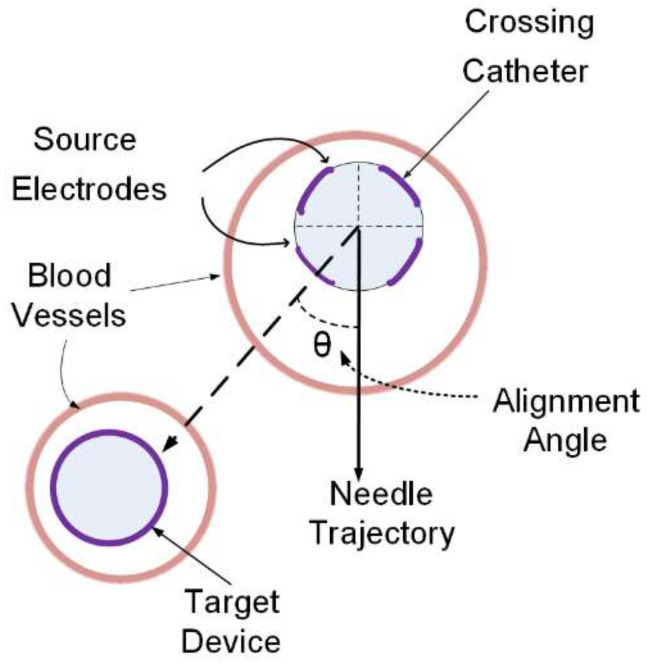
Arrangement of two catheters in the vessels.

To achieve the diversion of blood from one vessel to another, two catheters are used, one in each vessel, and these are termed the crossing catheter and the target device. Figure 1 depicts the arrangement; for clarity, it is depicted with a non-zero alignment angle, so, as shown, the needle will miss the target vessel. The crossing catheter is a 1.66 mm (5 French) diameter polymer tube and houses a sharp, hollow 0.6 mm diameter needle preformed with a 20 mm radius to bend away from the crossing catheter once ejected. The crossing catheter has a slot for the needle exit, and, distal to the exit slot, four identical, rectangular 3.5 mm long × 0.5 mm wide gold source electrodes are printed on the catheter tubing, equally spaced around the circumference. The gap between one pair of source electrodes is aligned to the needle exit slot. The electrode tracks are connected via a printed conductive track to a plug at the proximal end of the crossing catheter which is then connected to an external display unit. The target device is a 0.46 mm (2 French) wire with a cylindrical sensing electrode at the distal tip formed by removing the polymer covering to reveal the stainless-steel core which is connected to a proximal connector.

**Figure 2:**
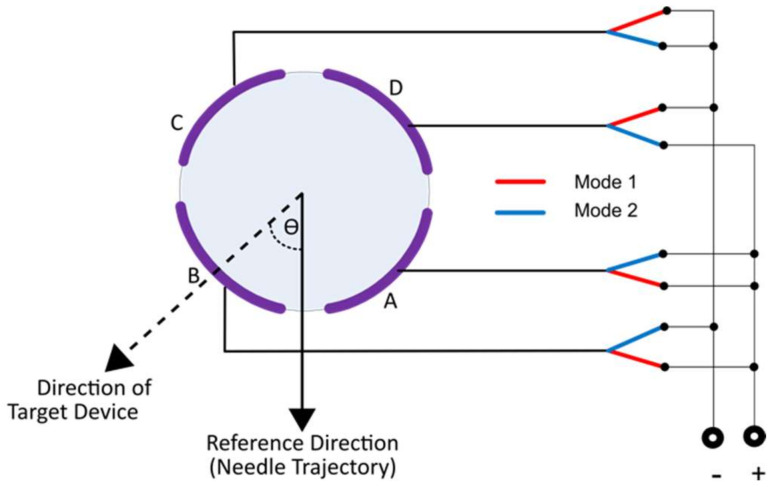
Connection to the source electrodes.

The source electrodes are in direct electrical contact with the blood or other conductive media. An AC voltage is applied to the source electrodes on the crossing catheter via a switch (Figure 2). In switch mode 1, the signal is applied between the front source electrodes relative to the needle exit slot (A&B connected) and the rear source electrodes (C&D connected). In switch mode 2, the signal is applied between the source electrodes left of the exit slot (B&C connected) and the source electrodes right of the slot (A&D connected). In either switch mode, the source electrodes form a dipole, and the voltage detected by the target device has a sinusoidal dependence on the angle ϴ between the direction of the target device and the needle exit slot.

**Figure 3:**
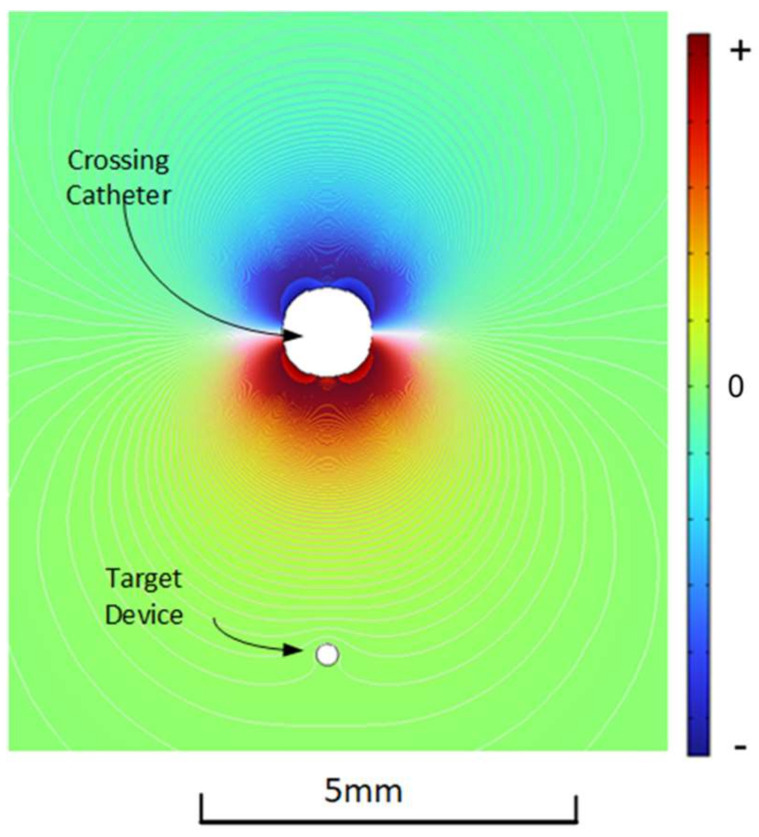
Spatial distribution of voltage amplitude generated.

Figure 3 shows the spatial variation of the voltage generated by the dipole in the plane of the source electrodes driven in mode 1. Voltages in front of the bottom two source electrodes are in phase with the driving voltage and are shown as positive (red), while voltages on the other side of the catheter are 180° out of phase and are shown as negative (blue). This was calculated using an EM simulation (AC/DC module COMSOL 6.0) via the frequency domain solver with an applied frequency of 25 kHz and a current of 1 mA per source electrode. The conductive electrodes were immersed in a 6 mS/cm conductive medium with values of electrical conductivity based on tissue values in the literature [10]. The mesh size was set to a range of 0.05 mm on the electrodes to 5 mm at the outer edge.

**Figure 4:**
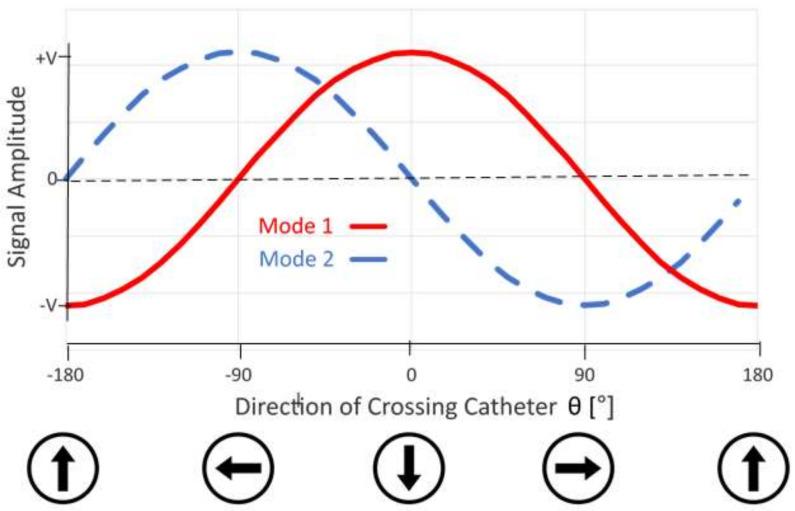
Angular dependence of received signal amplitude at a constant distance from the crossing catheter as a function on alignment angle relative to the target device. Mode 1: driven in “front–back” mode. Mode 2: driven in “left–right” mode.

The voltage has maximum amplitude adjacent to the bottom two (positive) source electrodes, and the amplitude is zero along the line that is equidistant from the top (negative) and bottom (positive) source electrodes where there is a phase inversion. At a fixed distance from the crossing catheter, the voltage detected by the sensing electrode has a sinusoidal dependence on the rotational angle of the crossing catheter relative to the needle exit point (Figure 4). This is referred to as the alignment angle ϴ when measured between the vector from the crossing catheter to the target device, and the vector corresponding to the direction that the needle exits the crossing catheter. Switching between a “front–back” dipole (mode 1) and a “left–right” dipole (mode 2) shifts the angular dependence by 90°. By having both measurements available, the angle from the reference point can be calculated using the arctan function; this is performed in the display unit.

**Figure 5:**
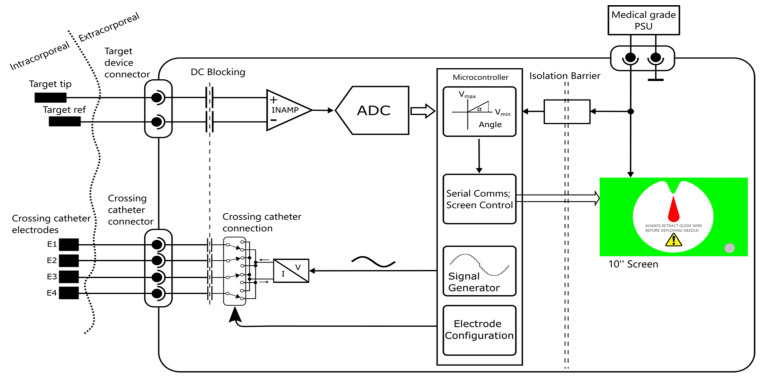
Block diagram of display unit.

The display unit generates a low voltage AC signal with a current within the limits set by EN60601-1 and with a fixed frequency in the kHz range which is alternately applied to the front–back or left–right electrode pairs, switching at 30 Hz. The signal from the sensing electrode on the target device is then amplified and digitised and its amplitude and phase extracted for each switch mode (Figure 5).

The angle ϴ between the reference direction and the target device is then calculated using the algorithm:

(1) $$\theta =arctan\left(\frac{Amplitude\;Mode\;1}{Amplitude\;Mode\;2}\right)$$

There is no discernible delay due to the calculation, and the angle is displayed in real time on a clockface display on a 10” screen. The alignment angle is continuously displayed whether the crossing catheter is stationary or being rotated. The clinician rotates the crossing catheter until the alignment angle ϴ is shown as 0° at the 12 o’clock position, indicating that the needle now points toward the target device and can be manually deployed. This physical rotation of the crossing catheter is similar to that performed in other percutaneous crossing procedures [5,6].

### 2.2. Vessel Diameters and Geometric Alignment Error in Vessel Positioning

The smallest vessels the devices are likely to be used for are in radiocephalic AVF creation. The minimum diameter of the radial artery is at least 2 mm, and a minimum cephalic vein diameter of at least 2 mm is also recommended to ensure suitable maturation and primary patency outcomes [11]. Therefore, vessel diameters of 2 mm can be considered the worst case for calculating the alignment accuracy. The target device will not necessarily be in the exact centre of the target vessel, but, in practice, it will be constrained to the central region of the vessel as it is delivered though a guide catheter which prevents it approaching the vessel wall.

**Figure 6:**
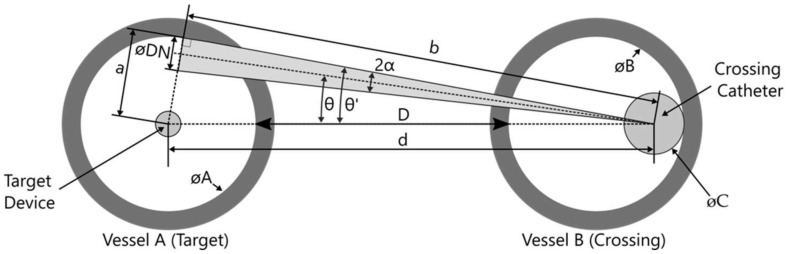
Geometric alignment error in vessel positioning.

Using basic trigonometry (Figure 6) and assuming the target device is in the vessel centre, the maximum alignment angle tolerance which still allows for a successful crossing (i.e., hitting a target vessel when deploying a needle from the first vessel) can be calculated using the equation:

(2) $$\sin\left(\theta^{\prime }\right)=\frac{a}{d}=\frac{\frac{\emptyset A}{2}}{\frac{\emptyset A}{2}+D+\emptyset B-\frac{\emptyset C}{2}}=\frac{\emptyset A}{\emptyset A-\emptyset C+2\left(D+\emptyset B\right)}$$

The needle diameter can be accounted for by subtracting *α* from *θ*′:

(3) $$\theta =\theta^{\prime }-\frac{1}{2}tan^{-1}\left(\frac{\emptyset DN}{b}\right)=\theta^{\prime }-\frac{1}{2}tan^{-1}\left(\frac{\emptyset DN}{\sqrt{d^{2}-a^{2}}}\right)$$

*θ*—angle tolerance (centre line of needle)

*θ*′—angle tolerance (edge of needle)

*d*—centre-to-centre distance between the target device and crossing catheter

*a*—radius of the target vessel lumen

*D*—tangential distance between vessel lumina

*DN*—needle diameter

*α*—needle centre line angle

∅*A*—diameter of the target vessel lumen

∅*B*—diameter of the crossing vessel lumen

∅*C*—crossing catheter diameter

The calculation is performed with the crossing catheter positioned at the rear of the first vessel B. This is realistic as the crossing catheter tends to be pushed back when the crossing needle is deployed and is also the worst case for alignment tolerance. For the worst case of 2 mm diameter vessels, an angle θ of 8° was calculated for an intra-catheter distance d of 5 mm. This means that the maximum alignment tolerance allowable is ±8° to ensure a successful needle crossing between two vessels. Using the same formula as above, crossing between two 3 mm vessels with an intra-catheter distance of 15 mm can be achieved with an alignment tolerance of ±4.2°.

The technique works in slightly curved vessels. The curvature of the crossing needle has been designed to intersect with the plane of the source electrodes at the typical separation of vessels, and, if a vessel is curved, then it will still intersect if the sensing electrode is approximately in the plane of the source electrodes. In the clinic, this is readily achieved using X-ray fluoroscopy. Furthermore, it is only the distal part of the catheters around the electrodes that needs to be reasonably parallel; the proximal length of the catheters can be curved.

### 2.3. Evaluation

**Figure 7:**
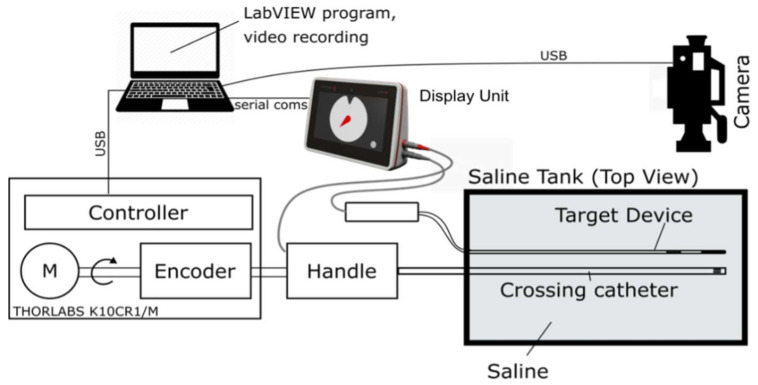
Saline bath test rig setup with cameras to verify alignment.

The performance of the guidance system was verified with two in vitro bench tests and in one ex vivo model. First, the crossing catheter and target device were mounted in a jig that permitted the crossing catheter to be rotated and the angle displayed on the screen with the alignment verified with a camera (Figure 7). The assembly was immersed in a tank filled with physiological saline. The crossing catheter was rotated by a stepper motor, and the display unit data were captured through a serial port and recorded in a csv file for postprocessing. The motor and the data capture were controlled through a LabVIEW program. The amplitude of the signals of the two modes were measured and the angle calculated using Equation (1).

A second in vitro evaluation was performed using conductive gel tissue phantoms which simulate the electrical properties of tissue. The gel was based on a recipe generally used for magnetic resonance imaging [12] to simulate the electrical properties of tissue (conductivity and permittivity) and consisted of 13.8 g of Tris buffer with 3.5 g of ammonium persulfate for a total quantity of 312.8 g of gel. The gel conductivity was measured using a conductivity meter with the electrodes immersed in the solution before and after setting. The conductivity level was chosen as it falls within the wide range of reported conductivities of human blood [13,14].

**Figure 8:**
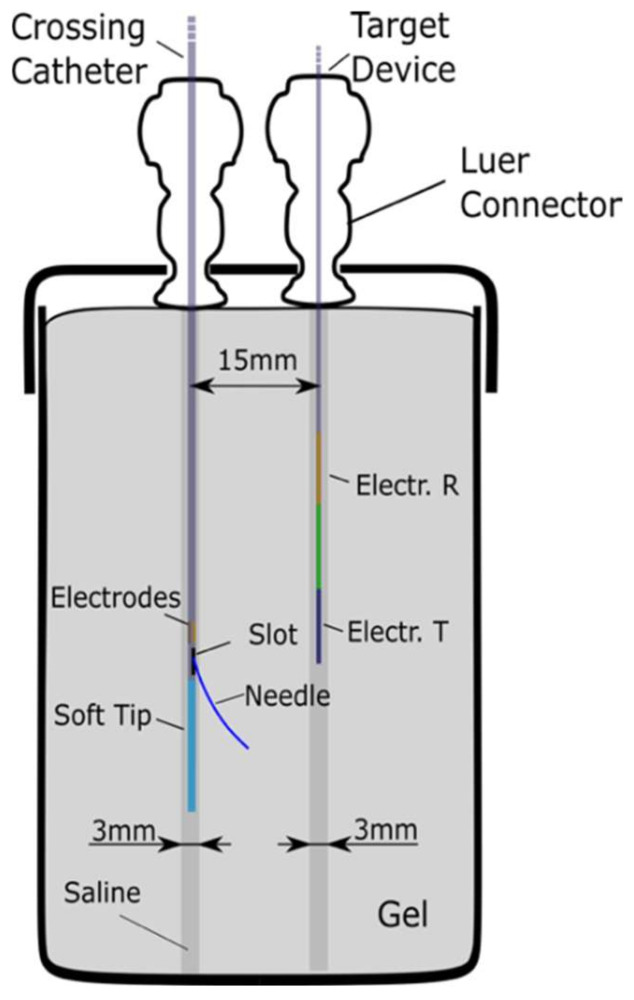
Schematic representation of the saline and gel phantom setup.

The crossing catheter was mounted in a 3 mm diameter tube formed in the gel matrix, and the target device was inserted into a neighbouring 3 mm diameter cavity (Figure 8). Both cavities, representing vessels separated by 15 mm, were filled with physiological saline (6 mS/cm).

**Figure 9:**
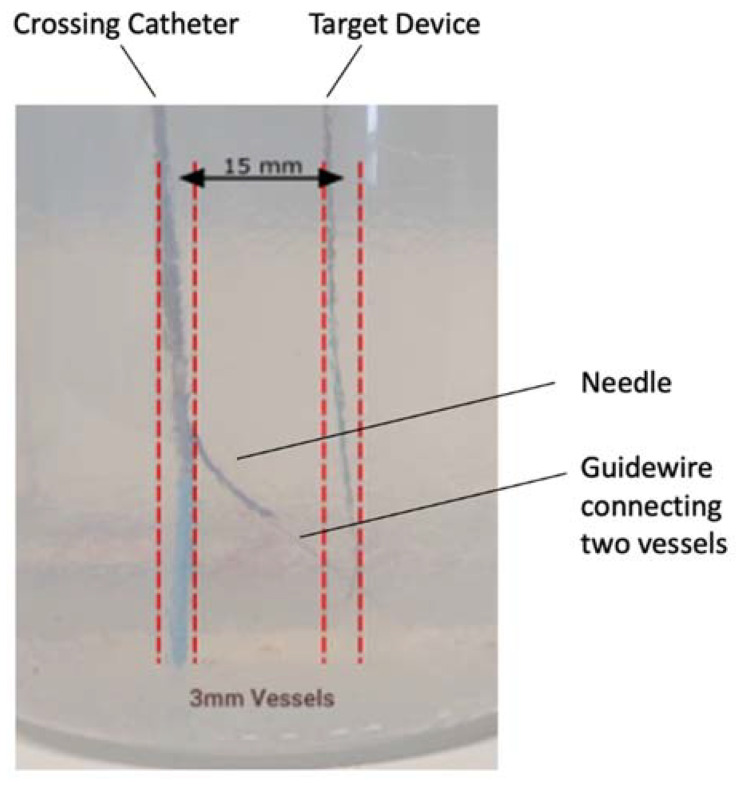
The gel phantom showing the needle deployed and the just discernible guide wire extended in the target vessel.

The ability of the display unit to guide the delivery of a needle from one vessel to another was demonstrated by aligning the crossing catheter relative to the target device using the indicator on the display only. The crossing catheter was rotated until the calculated alignment angle was 0° relative to the target device. The needle was then deployed and its final position observed; a guide wire was also inserted though the needle to check that it entered the target cavity (Figure 9). This was repeated 5 times.

Finally, the performance of the guidance system was tested in a commercially acquired ex vivo porcine tissue sample doused in saline solution to emulate human tissue properties. Two parallel cuts a few millimeters deep were made in the meat 10 mm apart to hold the catheters; these were doused with physiological saline and then covered with a flap of the tissue to fully surround the catheters. The crossing catheter was rotated and aligned on the display unit screen before deploying the needle. The top flap was then removed and the position of the needle tip relative to the target device noted. The alignment and needle deployment procedure was repeated 5 times and the position of the needle noted.

## 3. Results

### In Vitro and Ex Vivo

**Figure 10:**
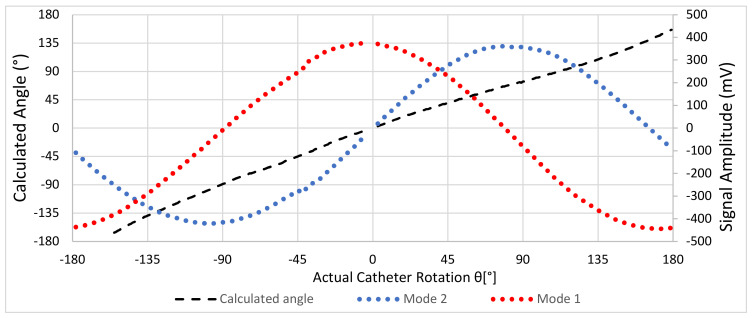
Measured signals and calculated alignment angle as a function of alignment angle in a saline bath.

The signals obtained as a function of an angle on a test tank filled with physiological saline are shown in Figure 10; this is the experimental equivalent of the EM-simulated values in Figure 4.

The signals from the two switching modes were perfectly sinusoidal and shifted by 90° as required, and the calculated (and displayed) angle corresponded to the actual crossing catheter alignment angle. The calculated angle was 0° when the alignment was 0°, and the needle was pointing directly at the target device and would, therefore, cross correctly. In addition, the relationship between the calculated angle and the actual angle was monotonic, so the user will always rotate in the correct direction when seeking alignment.

Tests in the gel phantom (as shown in Figure 9) found that the needle always entered the 3 mm diameter target cavity at a range of 15 mm. Finally, the test in the ex vivo tissue sample showed that the needle could be correctly deployed toward the target device based only on the alignment information displayed on the display unit. The needle deployment was verified by visual confirmation that the needle had touched the target device, and this was repeated successfully five consecutive times. As discussed in Section 2.2, this corresponds to an alignment accuracy of at least ±4°.

## 4. Discussion

The results presented in this paper do not completely reproduce the clinical situation, and there are some areas for further research to be addressed. First, real tissue is not homogeneous and will have components with different conductivities and permittivities which, in principle, could refract the electric fields so the line of zero field is not aligned with the needle trajectory. The homogeneous assumption is reasonable as there is no bone in the intervening tissue, so differences in conductivity are small. Furthermore, the different intervening tissues in the short distance between the catheters are generally symmetrical around the crossing trajectory, and, therefore, any refraction will not impact the calculated angle. Second, the linear alignment of the source and sensing electrodes was achieved visually as the phantoms were transparent; in the clinic, this will be performed using fluoroscopic guidance, and preliminary investigations indicate that the two techniques are similar. Third, the evaluation was performed with the catheters parallel, and, although the technique should work in slightly curved vessels, as discussed in Section 2.2, this is an issue to explore clinically. In practice, most vessels of interest in this technique are in limbs and are substantially parallel. Hence, further work is required to evaluate the performance of the technique in the clinical situation, in living tissue with pulsatile flow, or with different target device configurations. A version of the system for re-entry procedures in chronic total occlusions was granted regulatory approval in Europe, and a preliminary clinical case was performed successfully [15]. Clinical studies are planned which will investigate the use of the technique in other clinical applications such as endovascular radiocephalic AVF creation for dialysis. The technique can, in principle, be used in any application that requires delivering a therapy from one catheter to a target, and other applications are being explored.

To support its use in clinical practice, the device will need to show it provides advantages over current standard of care. When considering AVF creation for dialysis, the conventional surgical approach has a high failure rate within 12 months, thus, incurring significant costs associated with re-treatment. Endovascular AVF creation minimizes vessel trauma and has shown promising outcomes with high technical success rates, low failure rates, and good usability for haemodialysis [4].

Endovascular devices have recently been developed for the creation of AVFs in the antecubital fossa where the target artery and vein are immediately adjacent. These have already shown strong health economic benefits due to the lower number of follow-on interventions required to maintain patency and lower mean costs within the first year compared to surgically created AVFs [16,17].

Endovascular AVF creation at the wrist level between the radial artery and cephalic vein (radiocephalic AVF) presents significant challenges since the radial artery and cephalic vein are not immediately adjacent, thus, requiring alignment technology. This radiocephalic site is currently the first-line option for surgical AVF creation as it provides an easy-to-cannulate vein for dialysis treatment and enables subsequent surgical AVF creation in more proximal sites if it fails.

Electric-field-based guidance could help overcome these challenges and enable endovascular radiocephalic AVF creation at the wrist with the corresponding health and economic benefits. Further work is planned to obtain the requisite approvals to perform clinical trials with this new technology.

## 5. Conclusions

Generating electric fields in a conductive media with electrodes mounted on an intravascular catheter creates an asymmetric voltage distribution that can be detected by a second, neighbouring catheter. This enables the first catheter to be accurately oriented to deliver a needle towards the second catheter in an adjacent blood vessel to permit the percutaneous creation of anastomoses between vessels. The technique was shown to permit accurate needle delivery in in vitro and ex vivo models.

## 6. Patents

The work in this manuscript led to the filing of two patent families, WO2016145202 ‘Surgical Device for the Percutaneous Creation of an Arteriovenous Fistula (AVF)’ and WO2019202339 “Apparatus for Orientation Display and Alignment in Percutaneous Devices”.
